# Elderly Tai Chi practitioners exhibit superior multisensory integration in a dual-task paradigm

**DOI:** 10.3389/fnhum.2026.1776363

**Published:** 2026-02-27

**Authors:** Weiyi Dong, Tianxin Zheng, Pinyun Wu, Tingting Li, Jiugen Zhong, Yajun Zhang, Yong Zhang

**Affiliations:** School of Medicine, Shaoxing University, Shaoxing, China

**Keywords:** dual task, multisensory integration, older adults, postural stability, Tai Chi

## Abstract

**Objective:**

This study aims to investigate whether elderly Tai Chi (TC) practitioners demonstrate enhanced multisensory integration when performing dual-tasking.

**Methods:**

Twenty-six TC practitioners (TCP) with at least 5 years of TC experience and 28 controls (TC Naïve) without TC experience were recruited for this cross-sectional study. Standing was employed as the postural task and serial subtraction as the cognitive task to explore dual-task standing stability in the modified Clinical Test of Sensory Interaction on Balance (mCTSIB), which involved increasing the balance challenge via sensory conflict. The Center of Gravity (COG) Sway Velocity was measured to evaluate standing stability in the mCTSIB.

**Results:**

There was no significant difference in standing stability between the TCP and TC Naïve groups in mCTSIB during single tasks. However, the TCP exhibited significantly lower COG Sway Velocity than the TC Naïves under dual-task conditions. In the TC Naïve group, the main effects of single/dual task (Task) and balance challenge level (Level) were significant, but the main effects of Task were not seen in the TCP group. Additionally, the TCP group showed a significant interaction effect between Task and Level.

**Conclusion:**

Elderly Tai Chi practitioners demonstrate enhanced dual-task standing stability when facing balance challenges.

## 1 Introduction

Falling is the second leading cause of injury-related mortality worldwide (World Health Organization, 2021). Annually, approximately 68.4 million people die from falls, with 80% of these deaths occurring in low- and middle-income countries. In China, falling incidents represent the leading cause of death among people aged 65 years and older, with a tendency for increasing mortality rates and disease burden among the elderly from 1990 to 2019 (Ye et al., 2021). This highlight falls as a pivotal public health challenge in the process of healthy aging. It is well known that the ability to maintain balance is closely associated with the occurrence of falls. Mechanistically, balance is achieved or maintained by integrating vision, vestibular and somatosensory inputs into the central nervous system, and ensuring the musculoskeletal system responds appropriately. The process of combining information from different senses or modalities to create a unified, coherent and stable perception is termed multisensory integration (Zhang et al., 2020; Wang A. et al., 2023). This efficient process is essential for maintaining postural stability.

Derived from the same principles as the Sensory Organization Test (SOT), which encompasses six sensory conditions, to evaluate the patient's capacity to utilize visual, vestibular, and proprioceptive stimuli effectively, as well as the patient's ability to suppress erroneous sensory information (Ford-Smith et al., 1995). Shumway-Cook and Horak developed the Clinical Test of Sensory Interaction on Balance (CTSIB), which consists of six balance conditions: standing on an unstable surface or a stable surface with eyes open or closed, or in a visual-conflict dome (Ford-Smith et al., 1995). The CTSIB has been modified to include only the following four conditions: (1) standing with eyes open on a firm surface; (2) standing with eyes closed on a firm surface; (3) standing with eyes open on a foam surface; and (4) standing with eyes closed on a foam surface (Boonsinsukh et al., 2020). The mCTSIB takes less time to administer than the original CTSIB due to the exclusion of the two dome conditions, and it has been extensively employed to evaluate multisensory integration in clinical practice (Wrisley and Whitney, 2004; Desai et al., 2010; Freeman et al., 2018; Antoniadou et al., 2020). Extensive research has confirmed that multisensory integration impairment accompanies aging (De Dieuleveult et al., 2017; Zhang et al., 2020). The brain's ability to suppress erroneous or redundant information that disrupts postural stability is diminished in elderly people when multiple sensory inputs conflict or become inaccurate, and its capacity to select and amplify reliable sensory inputs is reduced (De Dieuleveult et al., 2017; Zhang et al., 2020). Mahoney JR et al. discovered that greater visual–somatosensory integration in older adults is associated with better balance and fewer falls (Mahoney et al., 2019). It was also revealed by the systematic review that impaired multisensory integration may increase the likelihood of falls in elderly people (Zhang et al., 2020). Furthermore, multisensory exercise engages multiple sensory pathways (e.g., visual, auditory, somatosensory and vestibular) simultaneously, improving older adults' multisensory integration and balance function while reducing the risk of falls (Merriman et al., 2015; Allison et al., 2018; Zhang et al., 2021). Consequently, multisensory integration is recognized as a significant potential “target” for fall risk intervention, which is an important consideration for healthcare providers.

In recent years, many multisensory exercise interventions have been devised to improve balance in older adults by enhancing the ability of the central nervous system to process and integrate sensory afferents, and by facilitating compensation for deficient sensory inputs. However, most of these interventions use new balance platforms and advanced equipment, such as the Balance Freedom™ headband system (Balance Innovations GmbH, Switzerland) and the SMART Balance Master (NeuroCom International, Inc., Clackamas, OR, United States), which are expensive commercial products and require special training to administer (Merriman et al., 2015; Allison et al., 2018; Zhang et al., 2021). Meanwhile, exergames have been developed to improve multisensory function, such as the Nintendo “Wii Balance Board” or “Wii Fit,” but these may be unsuitable for older adults as the commercially available games do not consider fall prevention (Merriman et al., 2015). Therefore, efficacious and universally applicable interventions targeting multisensory integration must be explored, to minimize the occurrence of falls in the elderly population. Tai Chi has been shown to be extremely efficient and economical in reducing the incidence of falls among the elderly. Tai Chi emphasizes the coordinated combination of eye concentration, hand positioning and postural equilibrium, which stimulates numerous sensory pathways, such as the visual, vestibular and somatosensory systems, making it a multisensory exercise (Wong et al., 2001; Lan et al., 2013). Previous studies have confirmed that long term Tai Chi practice improved multisensory integration in older adults, as well as exhibiting superior postural stability under sensory conflict (Wong et al., 2001; Tsang and Hui-Chan, 2004; Tsang et al., 2004; Wang A. et al., 2023; Cui et al., 2024). However, the above findings were predominantly derived from single-task conditions, and in real-life scenarios, elderly individuals frequently perform routine activities in multi-tasking environments, such as walking and talking. Currently, there is a paucity of reports on the effects of Tai Chi on multisensory integration in dual-task conditions among the elderly, which may limit the scalability of such balance training interventions.

Additionally, the dual-task paradigm is commonly used to investigate the allocation and shifting of attentional resources. According to Cognitive Load Theory, the brain has a limited capacity to analyse and process information. When cognitive and postural tasks are performed concurrently, they compete for attentional resources, generating a cognitive-motor interference effect, which consequently results in diminished cognitive performance or postural stability (Yeh et al., 2019; Bayot et al., 2020). Furthermore, as task difficulty increases and available cognitive resources decrease in dual-task conditions, it may be necessary to switch attention between tasks depending on their priority to successfully complete one of them (Howard et al., 2017; Kan et al., 2025). Typically, prioritization is determined by the motivation to minimize danger and maximize pleasure (Yogev-Seligmann et al., 2012). Sacrificing performance on a task in pursuit of maintaining balance is known as the “posture first strategy” (Howard et al., 2017; Johansson et al., 2021; Kan et al., 2025). Conversely, the “posture second strategy” involves sacrificing postural control to perform secondary tasks, thereby increasing the risk of falling (Howard et al., 2017; Johansson et al., 2021; Kan et al., 2025). Researchers have identified that cognitive-motor interference increases with age, cognitive and sensorimotor impairments, and that there is a predisposition for “posture second strategy” (Yogev-Seligmann et al., 2012; Tetsuya Ohzuno, 2019; Castelli et al., 2021). Extensive evidence indicates that Tai Chi practice could reduce cognitive-motor interference effects in older adults, thereby optimizing the allocation of attention resources and ensuring postural stability (Lu et al., 2013, 2016; Wayne et al., 2015; Varghese et al., 2016; Song et al., 2018; Li et al., 2021). Therefore, researchers hypothesized that elderly Tai Chi practitioners exhibit enhanced postural stability when dual-task requirements become more challenging, which is in accord with a “posture first strategy” phenomenon. Thus, the dual-task paradigm was used to ascertain balance performance in elderly Tai Chi practitioners using the mCTSIB in our study.

## 2 Materials and methods

### 2.1 Participants

A total of 54 older adults (aged 50–79 years) were recruited through the Shaoxing Senior Citizens' Sports Association and the Shaoxing Senior Citizens' University. Among them, 26 were Tai Chi practitioners (seven males and 19 females, average age: 65.46 ± 4.68 years) who had practiced Yang-style Tai Chi for a minimum of three 30-min sessions per week, undertaken for a period of more than 5 years (average Tai Chi experience: 14.08 ± 8.46 years). The remaining 28 participants had no Tai Chi training experience and maintained their daily physical activity habits (nine males and 19 females, average age: 65.89 ± 7.89 years), constituting the Tai Chi Naïve group (TC Naïve). The exclusion criteria for both groups included the following instructions: (1) unstable cardiovascular conditions, such as myocardial infarction, angina pectoris or uncontrolled arrhythmia; (2) neurological disorders, including stroke, multiple sclerosis, Parkinson's disease or peripheral neuropathy; (3) history of cancer surgery within the past 5 years; (4) hypertension or diabetes, with poorly controlled blood pressure or blood glucose levels; (5) musculoskeletal disorders presenting marked symptoms of pain in the neck, shoulder, back or legs; (6) hospitalization within the past 6 months for an acute illness; (7) self-reported risk of falling during ambulation, or alcohol abuse; and (8) mini-mental state examination (MMSE) score below 24.

### 2.2 Experimental design

#### 2.2.1 Procedures

After completing the physical examination and the MMSE, the subjects underwent the mCTSIB with or without a cognitive task (dual-task or single-task) in a randomized order. It is stated that the 15-min break between the balance tasks and the cognitive condition was used as a washout period to prevent interference between the different tests (Varghese et al., 2016). During this period, the participants in our study should complete the International Physical Activity Questionnaire (IPAQ), which assessed their daily activity habits and physical activity levels over the previous week. Therefore, a minimum interval of 30 min was required between each assessment. According to the IPAQ categories for health, as defined by the IPAQ scoring protocol (www.ipaq.ki.se), these levels were categorized as follows: heavy, moderate or light (Fogelholm et al., 2006). This experimental protocol was approved by the Academic and Ethical Review Committee at Shaoxing University (USX-RTYJ-20250501). All participants were informed of the potential risks and benefits of taking part in this study, and confirmed their voluntary participation by signing an informed consent form following the standards of the Declaration of Helsinki.

#### 2.2.2 Cognitive task

We used the Serial Subtractions cognitive task, in which the subjects performed mental arithmetic while undergoing mCTSIB. The researchers instructed the participants to try their best to complete both the postural stability and serial subtraction tasks without providing specific instructions on task prioritization. Before each test, the subjects were given a three-digit number (any figure between 490 and 500) and told to quickly subtract 3 from this number, while reporting the results out loud. The number of correct responses generated within the 10-s duration of each condition in mCSITB was recorded.

#### 2.2.3 Outcome measures

The center of gravity sway velocity (COG Sway Velocity) was measured via the mCTSIB using a long force plate balance platform (Balancemaster, Neurocom^®^). The mCTSIB comprises four tests arranged in ascending order of difficulty for postural control: Firm Surface with Eyes Open (Firm-EO), Firm Surface with Eyes Closed (Firm-EC), Foam Surface with Eyes Open (Foam-EO) and Foam Surface with Eyes Closed (Foam-EC). Each protocol was performed three times consecutively, with each test lasting 10 s. The mean COG Sway Velocity was obtained under each of the four test conditions. The greater the COG Sway Velocity, the poorer the postural stability. Sufficient rest periods were scheduled between each procedure. Throughout testing, subjects were required to maintain a stable standing posture.

#### 2.2.4 Statistical analysis

Based on the results of related study (Wang et al., 2024), it was determined that a sample size of 18 participants per group would achieve 80% statistical power at an alpha level of 0.05 when analyzing differences between groups in standing balance under reduced sensory conditions. Thus, it was considered that 26 subjects for each group would be adequate for the objective of the current study. All data were processed using SPSS 25.0 software (IBM SPSS Statistics, SPSS Inc., Chicago, IL, United States). Normality was assessed using a combination of the Shapiro–Wilk test and graphical analysis. Q-Q plots showed data points mostly along the reference line and histograms had near-bell-shaped curves, which supports the normal distribution. For ANOVA degrees of freedom failing sphericity tests, the Greenhouse–Geisser correction was applied. The age, height, weight, and MMSE scores of the participants are shown as the mean ± standard deviation (*M* ± SD). Differences in these metrics between the TCP and TC Naïve groups were assessed using independent samples *t*-tests, whilst gender, education distribution and physical activity level classification composition differences between groups were analyzed via chi-square tests. Statistical significance was indicated by *p* < 0.05.

First, to examine whether elderly individuals with long-term Tai Chi practice exhibit superior postural control when responding to sensory conflict, a 2 × 4 mixed-design ANOVA was conducted with group (TCP and TC Naïve) as the between-subject factor and balance challenge level (Firm-EO, Firm-EC, Foam-EO, and Foam-EC) as the within-subjects factor under single-task and dual-task conditions. The effect of raising the balance challenge was established by the main effect of Level and the differential reaction of the two groups was indicated by the Group × Level interaction or the main effect of Group. Second, to further clarify the tendency of postural strategies employed by the two groups of subjects when confronted with increased balance challenges under dual-task conditions, a within-group 2 × 4 repeated-measures ANOVA was used for both TCP and TC Naïve. The within-subjects variables were task type (single-task or dual-task) and balance challenge level (Firm-EO, Firm-EC, Foam-EO, and Foam-EC). If there was a significant Task × Level interaction consistent with a smaller increase in dual-task sway compared to single-task sway with increasing balance challenge, suggesting the “posture first strategy” phenomenon. The “posture second strategy” phenomenon was implicated by a significant main effect of Task or Task × Level interaction due to a greater sway in dual-task vs. single task (Howard et al., 2017). A significance level of α = 0.05 was set, and multiple *t*-tests with Bonferroni corrections were used for follow-up comparisons if the results of the ANOVA showed significant main effects or interactions.

## 3 Results

### 3.1 Participant characteristics

As shown in Table 1, there were no significant differences between the TCP and the TC Naïve groups with regard to age, height, weight or MMSE scores. No significant differences were also found in gender composition, physical activity level or education distribution between the two groups.

**Table 1:** Demographics of the TCP and the TC Naïve participants.

**Characteristics**	**TCP (*n* = 26)**	**TC Naïve (*n* = 28)**	***p*-value**
Age (yeas)	65.46 ± 4.68	65.89 ± 7.89	0.810
Height (cm)	160.73 ± 5.75	160.89 ± 7.14	0.928
Weight (kg)	59.44 ± 6.39	59.55 ± 7.65	0.954
**Sex (** ***n*** **)**			
Male/female	7/19	9/19	0.770
**Physical activity level (** ***n*** **)**			
Heavy/moderate/light	15/10/1	18/10/0	0.549
**Education (** ***n*** **)**			
Primary/secondary/university	5/19/2	9/14/5	0.211
MMSE score	27.50 ± 2.08	27.00 ± 2.48	0.428

### 3.2 Standing stability when addressing balance challenges under single/dual task conditions

As shown in Table 2, under the single-task condition, there was no main effect of group (*F*_(1, 52)_ = 0.443, *p* = 0.509, η^2^ = 0.008), while the main effect of balance challenge level was significant (*F*_(1.7, 87.3)_ = 552.7, *p* < 0.01, η^2^ = 0.914); furthermore, the interaction between group and balance challenge level was not significant (*F*_(1.7, 87.3)_ = 0.485, *p* = 0.584, η^2^ = 0.009). Under the dual-task condition, the main effects of group (*F*_(1, 52)_ = 13.708, *p* = 0.001, η^2^ = 0.209) and balance challenge level (*F*_(2.0, 105.5)_ = 232.77, *p* < 0.001, η^2^ = 0.817) were both significant, but the interaction between group and balance challenge level was not significant (*F*_(2.0, 105.5)_ = 0.776, *p* = 0.465, η^2^ = 0.817). Following Bonferroni correction, a significantly higher TCP group (*M* = 0.917, SE = 0.035) was found compared to the TC Naïve group (*M* = 1.097, SE = 0.034, *p* = 0.001).

**Table 2:** COG sway velocity in the mCTSIB under single/dual task conditions.

**Task**	Balance challenge level			
	**Firm-EO (deg/s)**	**Firm-EC (deg/s)**	**Foam-EO (deg/s)**	**Foam-EC (deg/s)**
**Single-task**			
TC Naïve	0.34 ± 0.06	0.40 ± 0.11	0.92 ± 0.30	2.00 ± 0.44
TCP	0.37 ± 0.07	0.38 ± 0.07	0.84 ± 0.20	1.95 ± 0.43
**Dual-task**			
TC Naïve	0.63 ± 0.25	0.58 ± 0.22	1.18 ± 0.38	2.00 ± 0.53
TCP	0.53 ± 0.21	0.44 ± 0.08^*^	0.95 ± 0.28^*^	1.75 ± 0.37^*^

^*^Indicates TCP vs. TC Naïve by independent t-test with Bonferroni corrections, p < 0.05.

### 3.3 Postural strategy prioritization in dual-task conditions with progressive balance challenge

**Figure 1:**
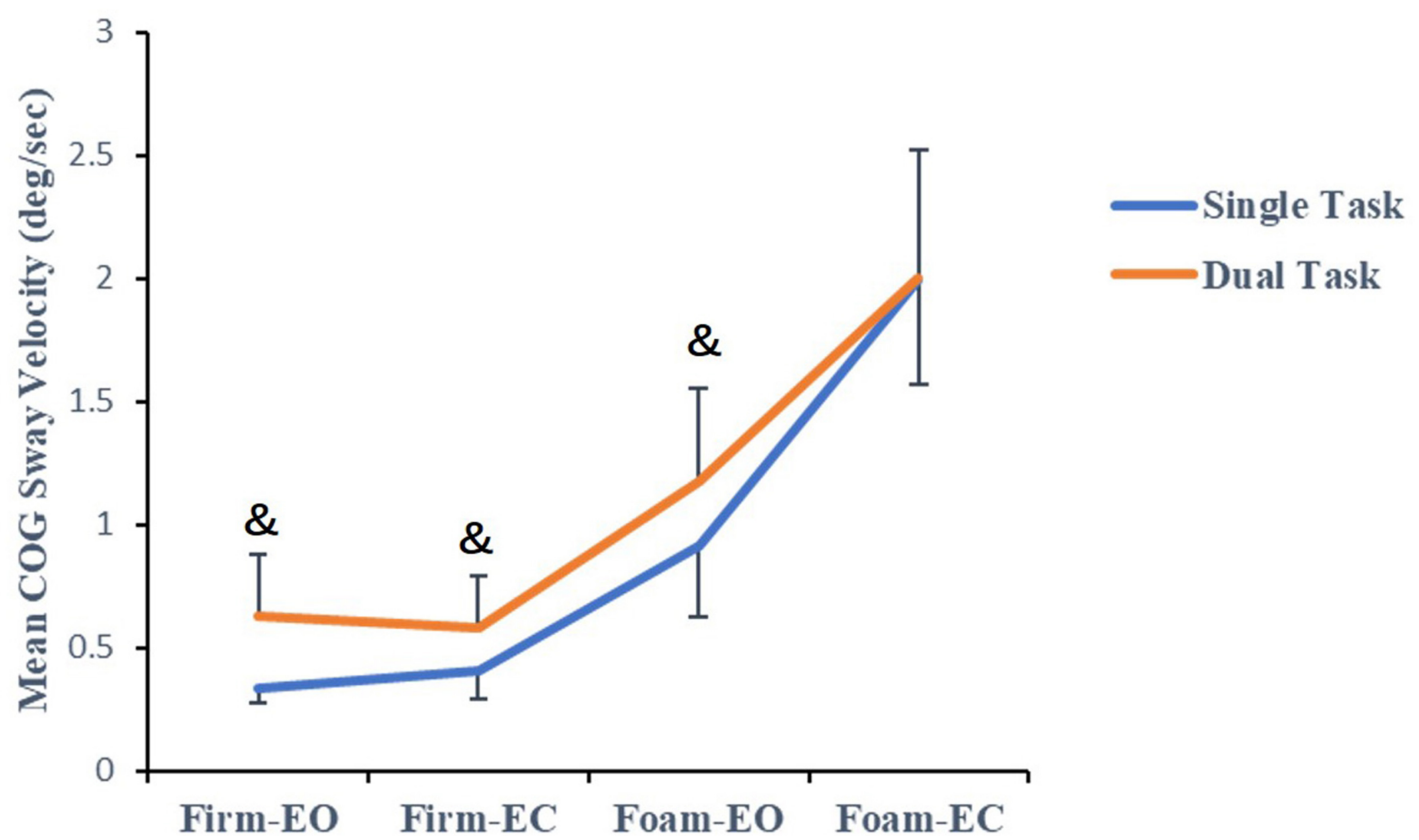
Mean COG sway velocity of the TC Naïve group in single/dual task (“&” indicates dual task vs. single task paired *t*-test, *p* < 0.05).

**Figure 2:**
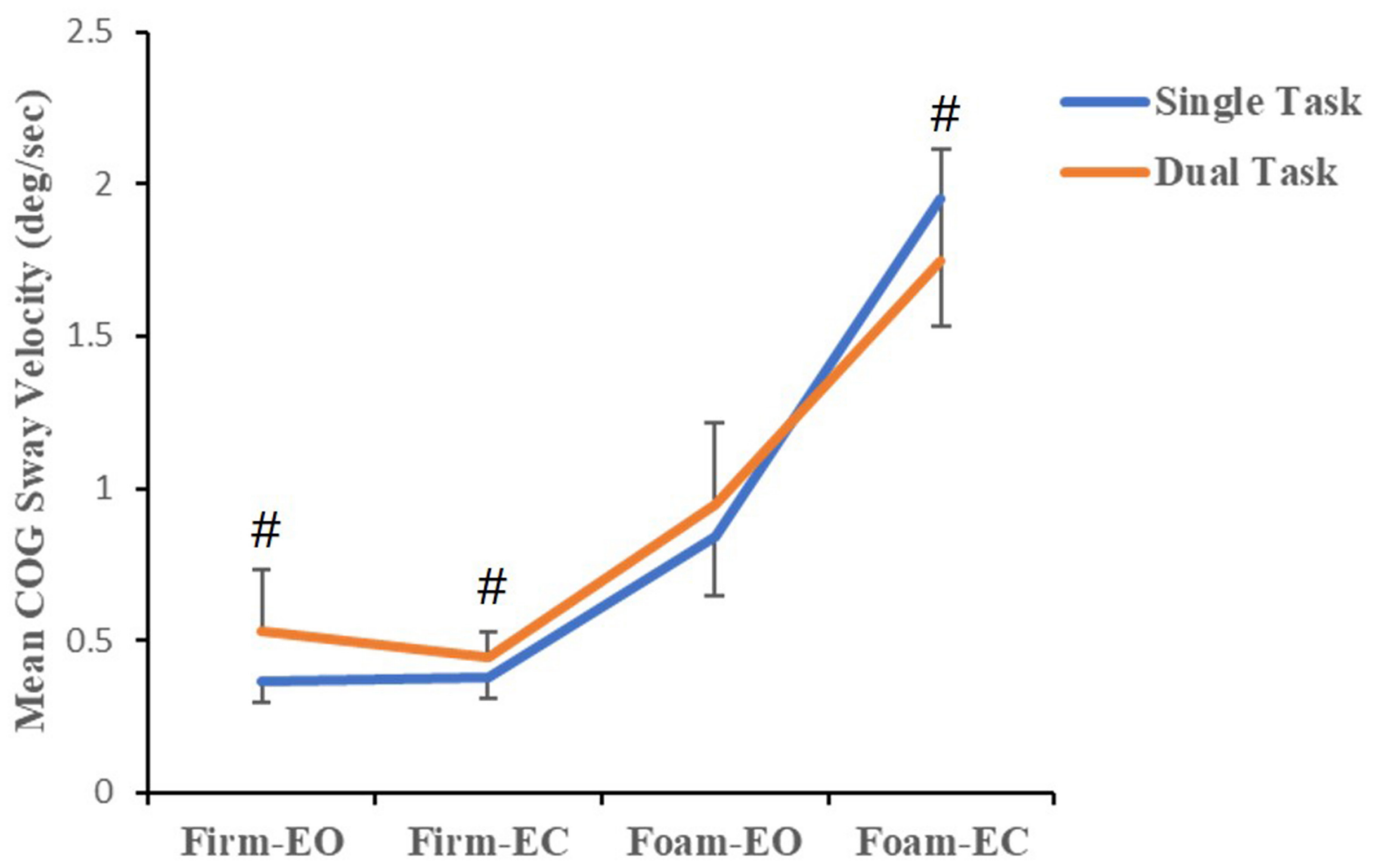
Mean COG sway velocity of the TCP group in single/dual task (“#” indicates dual task vs. single task paired *t*-test, *p* < 0.05).

The TC Naïve group (see Figure 1) showed significant main effects for both Task (*F*_(1, 54)_ = 12.671, *p* = 0.001, η^2^ = 0.190) and Level (*F*_(1.93, 104)_ = 320.05, *p* < 0.001, η^2^ = 0.856), though no significant interaction between Task and Level was observed (*F*_(1.93, 104)_ = 2.634, *p* = 0.079, η^2^ = 0.047). As shown in Figure 2, the TCP group exhibited a non-significant Task main effect (*F*_(1, 50)_ = 0.686, *p* = 0.411, η^2^ = 0.014). However, there was a significant Level x Task interaction (*F*_(1.74, 86.91)_ = 5.845, *p* = 0.006, η^2^ = 0.105) and a significant Level main effect (*F*_(1.74, 86.91)_ = 408.414, *p* < 0.001, η^2^ = 0.891). Furthermore, the TC Naïve group exhibited higher COG Sway Velocity under dual-task conditions compared to single-task conditions in the Firm-EO, Firm-EC and Foam-EO tests (*p* < 0.05), as did the TCP group, but only in the Firm-EO, Firm-EC tests. Conversely, the TCP group demonstrated lower COG Sway Velocity during dual-task conditions in the Foam-EC test relative to single-task conditions (*p* < 0.05).

## 4 Discussion

The dual-task paradigm is commonly used to investigate how attention is allocated across multiple concurrent tasks. It also allows us to explore how participants prioritize tasks when the difficulty of either the cognitive or postural task increases. We ascertained that the TCP group demonstrated better balance performance in the mCTSIB test under dual-task conditions, exhibiting superior multisensory integration in comparison to the TC Naïve group. As the posture task became more challenging, the TCP group demonstrated a “posture first” phenomenon, whereas the TC Naïve group exhibited a “posture second” phenomenon.

The capacity of elderly individuals to effectively manage environmental challenges and maintain equilibrium is reliant on the brain's process of analyzing and integrating multimodal sensory input. Impairment in this multisensory integration capacity, which is common with age, frequently contributes to falls in older adults (De Dieuleveult et al., 2017; Zhang et al., 2020). Research confirms that long-term Tai Chi practice improves multisensory integration capacity in older adults, as demonstrated by enhanced postural stability during sensory conflict in single-task SOT tests (Wong et al., 2001; Tsang and Hui-Chan, 2004; Tsang et al., 2004; Wang A. et al., 2023). Nevertheless, our study, employing the mCTSIB test, found no postural stability advantage in Tai Chi practitioners, potentially as a consequence of differing multisensory integration assessment methodologies. In the aforementioned Tai Chi-related studies, sudden anterior-posterior movements of the standing platform were primarily employed to induce postural disturbance in tests of SOT. This approach mainly activates bottom-up compensatory postural adjustments via feedback mechanisms to maintain standing stability, thereby increasing the difficulty of postural control (Piscitelli et al., 2017). In contrast, the mCTSIB test uses a sponge mat as an unstable support surface (Desai et al., 2010; Boonsinsukh et al., 2020). The testing process primarily relies on subjective psychological expectations generated, through vision and other sensory channels, to trigger feedforward control, which enables anticipatory postural adjustments to be made efficiently to maintain balance (Togo and Imamizu, 2016). Therefore, it is considered that the difficulty of maintaining postural stability during the mCTSIB test is lower than that of the SOT test. Furthermore, due to inherent differences in balance function across populations, individuals with impaired balance may exhibit a “floor effect” in SOT testing, while healthy individuals may demonstrate a “ceiling effect” in mCTSIB testing (Murray et al., 2014). In response, Freeman L et al. discovered that mCTSIB testing could distinguish standing stability differences between elderly groups with and without a history of falls, whereas no such differences were observed in the more challenging SOT test (Freeman et al., 2018). In contrast, the SOT test may exhibit higher sensitivity when applied to healthy individuals or groups with superior balance capabilities (Murray et al., 2014). Tsang WW et al. also observed that elderly Tai Chi practitioners demonstrated significantly superior balance performance in the SOT test compared to healthy age-matched controls, approaching levels comparable to those of healthy young adults (Tsang et al., 2004). It is clear that both the SOT and mCTSIB tests can assess multisensory integration capability by evaluating standing stability under sensory conflict conditions, but the two tests differ in their complexity, which may also reveal differences in sensitivity between groups, necessitating consideration of subjects' inherent balance function characteristics in practical applications.

Evidence indicates that older adults or individuals with impaired balance function experience heightened difficulty in postural control when performing posture tasks combined with cognitive activities, even leading to diminished performance in one or both tasks, a phenomenon known as cognitive-motor interference (Bayot et al., 2020). However, Tai Chi practice reduces cognitive-motor interference effects in older adults, enabling them to effectively achieve automatic balance control, reduce gait parameter variability, and maintain postural stability during stair descent even under dual-task conditions (Lu et al., 2013, 2016; Wayne et al., 2015; Varghese et al., 2016; Song et al., 2018; Li et al., 2021). Unfortunately, few studies have examined the effects of Tai Chi practice on multisensory integration in older adults under dual-task conditions, using the mCTSIB/SOT as a postural task. In real life, however, people often have to deal with balance challenges caused by sensory conflicts in dual-task or multiple-task situations. Currently, only Hall CD et al. reported that a 12-week Tai Chi intervention (twice weekly, 1.5 h per session) had no beneficial effects on standing stability when older adults concurrently performed an auditory discrimination task and the SOT test (Hall et al., 2009). This may be because the dual-task paradigm is not sensitive or specific enough to evaluate the effects of Tai Chi in older adults. Research indicates that appropriate cognitive load is key for effectively constructing dual-task paradigms (Yeh et al., 2019; Kan et al., 2025). If the cognitive load is too low, it struggles to induce cognitive-motor interference; if the cognitive load is too high, it may lead to experimental failure (Reed et al., 2022). AI-Yahya et al. found through a systematic review that exogenous attention (e.g., auditory discrimination tasks) produces weaker cognitive posture interference effects than endogenous attention (e.g., serial subtraction) (Al-Yahya et al., 2011). Using auditory discrimination tasks as a cognitive load in dual-task paradigms may not be sensitive enough to evaluate the effects of a Tai Chi intervention on dual-task performance in older adults. Furthermore, Kim et al. reported that Tai Chi practice primarily enhanced cognitive function in older adults through endogenous attention and executive function (Kim et al., 2016). Selecting exogenous attention as the cognitive load in dual-task conditions for evaluating Tai Chi intervention efficacy may fail to meet the specificity requirements. Regarding the above-mentioned issues, studies on Tai Chi reducing cognitive posture interference have predominantly used the “serial subtraction” cognitive load in the dual-task paradigms (Wayne et al., 2015; Song et al., 2018; Li et al., 2021).

The dual-task paradigm employed in this study also used “serial subtraction” to increase the difficulty of the postural control during the mCTSIB testing process. The results showed that the TC Naïve group had significantly poorer standing stability than the TCP group in the Firm-EC, Foam-EO and Foam-EC tests. However, there was no significant difference between the two groups in the Firm-EO test, which was the simplest balance challenging test. These results suggest that elderly Tai Chi practitioners can efficiently manage balance challenges resulting from visual and proprioceptive interference in dual-task scenarios, exhibiting superior multisensory integration capabilities. Consistent with existing reports, this may relate to the benefits of Tai Chi practice for elderly individuals, involving sensory reweighting and cognitive function (Lu et al., 2013; Kim et al., 2016; Chen et al., 2022; Cui et al., 2024; Liu et al., 2024). Sensory reweighting is the process by which the brain determines the weighting applied to information from different sensory channels based on their reliability (Allison et al., 2018; Cui et al., 2024; Liu et al., 2024). However, older adults usually experience greater postural instability during sensory conflict than young adults, due to inefficient and inflexible sensory reweighting (Allison et al., 2018; Cui et al., 2024; Liu et al., 2024). Research indicates that older Tai Chi practitioners show a relatively faster sensory processing and higher flexible sensory reweighting, which prioritizes reliance on reliable sensory input while effectively suppressing unreliable or disrupted sensory information when confronting sudden balance disturbances (Allison et al., 2018; Cui et al., 2024; Liu et al., 2024). Additionally, it was reported by Kim et al. that mental-attentional vigilance in older adults can be improved by Tai Chi, with attentional control being strengthened, enabling swift and accurate selection of the target task, and facilitating efficient cognitive resource allocation when processing distracting information (Kim et al., 2016). Consequently, our results suggest that the older Tai Chi practitioners may retain an advantage in sensory reweighting under dual-task conditions, thereby effectively maintaining postural stability when confronting sensory conflicts.

Our study further revealed that both the TCP and the Naïve groups exhibited significantly reduced standing stability during the Firm-EO and Firm-EC tests combining cognitive load (which presented the lowest balance challenge) compared to their respective single-task conditions. This suggests the existence of a cognitive-motor interference effect, consistent with prior dual-task research findings. However, as the balance disturbance increased, the TCP group demonstrated enhanced dual-task standing stability in Foam-EO test, reaching the single-task level. The TC Naïve group remained markedly below the single-task performance level. In the Foam-EC test involving a cognitive task, which presented the greatest balance challenge, standing stability showed an upward trend in both groups, with the TCP group achieving significantly better performance than their own single-task baseline. Similar findings were reported by Yeh TT et al., who found that high cognitive load facilitated dual-task standing stability in adolescent gymnasts, but hindered performance in the general control group (Yeh et al., 2019). Yeh TT et al. also proposed that the SOT test may be relatively simple for adolescent gymnasts, whereas the general control group encountered difficulties, and moreover, through prolonged training, adolescent gymnasts achieve automated postural control, reducing cognitive resource demands for posture tasks, whilst the additional cognitive load optimizes arousal levels, facilitating optimal postural adjustments (Yeh et al., 2019). Conversely, the general control group faced increased cognitive demands when combining progressive postural disturbances, which further intensified competition for limited central resources and thereby impeded improvements in balance performance. This finding is in concurrence with the inverted U-shaped theory of posture performance and arousal levels (Huxhold et al., 2006), which may elucidate why Tai Chi practitioners exhibited enhanced postural stability in our study when confronted with balance challenges under dual-task conditions. Additionally, the Foam-EC test, which presented the greatest balance challenge, favored improved postural stability in dual tasks, particularly in the TCP group. By summarizing previous research (Yeh et al., 2019; Kan et al., 2025), this may be related to the brain's strategy for prioritizing tasks when competing for attention resources in a cognitive-motor dual-task paradigm. In situations with higher risk of falling, individuals prioritize maintaining balance even at the expense of cognitive performance, which indicates the “posture first” phenomenon.

Furthermore, the dual-task paradigm was found to enhance postural stability in the TCP group when confronted with balance challenges, indicating a tendency toward a “posture first” phenomenon. However, this phenomenon was not observed in the TC Naïve group, whose balance performance declined under the dual-task condition, consistent with a “posture second” phenomenon. It is still unclear how the central nervous system decides which tasks to prioritize under dual-task conditions, particularly during the cognitive load or balance disturbance difficulty arising. Researchers have been suggested that this may result from a trade-off between an individual's perception of fall risk and the task priority (Yogev-Seligmann et al., 2012; Howard et al., 2017; Kan et al., 2025). When attention resources are sufficient for the dual-task demands without inducing cognitive-motor interference, the “posture-second strategy” is adopted, especially the postures are not too difficult and the risk of falling is low (Howard et al., 2017; Johansson et al., 2021; Kan et al., 2025). However, as postural task difficulty increases and fall risk emerges, they shift to a “posture-first strategy” (Howard et al., 2017; Johansson et al., 2021; Kan et al., 2025). Notably, our study observed pronounced cognitive-motor interference effects in both participant groups during the Firm-EO test, which presented the lowest balance disturbance, indicating the absence of the aforementioned “posture second” phenomenon. Moreover, other studies report that certain populations with impaired somatosensory functions (such as some elderly individuals, those with Parkinson's disease, stroke or multiple sclerosis) continue to adopt the “posture-second strategy,” and even when fall risks exist, they still sacrifice postural stability to prioritize allocating attentional resources to secondary tasks (Corp et al., 2018; Huang et al., 2018; Castelli et al., 2021; Johansson et al., 2021). This may be connected to age-related or central nervous system diseases leading to working memory reduction and negatively impacting brain decision-making processes, thus hindering the rationality of fall risk perception in these groups. Similarly, the TC Naïve group may also exhibit the “posture second” phenomenon for this reason in the study. Further evidence suggests that Tai Chi practice can enhance working memory in elderly individuals (Kim et al., 2016; Wang C. et al., 2023) and that Tai Chi practitioners demonstrate superior fall risk perception when confronted with balance challenges (Liu et al., 2024), which may enable attentional resources to be prioritized for postural stability in dual tasks. Furthermore, the process of aging is frequently accompanied by a decline in postural stability when executing motor-cognitive activities. It has been proposed that this is connected to decreased task-related brain activity in older people in the prefrontal cortex (PFC) and the primary motor cortex (M1), as well as to reduced disinhibition of PFC-M1 pathways during movement preparation (Corp et al., 2018). Consequently, a “posture second” prioritization strategy was adopted. In contrast, older adults who displayed better performance in motor-cognitive tasks have been shown to increase PFC activity (Kan et al., 2025). Recent evidence has demonstrated that Tai Chi practice increases bilateral activation of the PFC to prioritize gait performance and optimizes the symmetry of functional connectivity in cortical regions (Chen et al., 2022, 2025). This suggests that Tai Chi may enhance neural adaptability and strengthen functional connectivity by promoting the formation of new neural network connections and activating brain regions related to motor control. Therefore, elderly Tai Chi practitioners in our study exhibited superior equilibrium when their balance was challenged under dual-task conditions, in line with the “posture first” phenomenon, possibly due to TCP neuroplasticity.

This study also presents several limitations. Firstly, the cross-sectional design merely observed that elderly Tai Chi practitioners demonstrated advantages in sensory integration and task prioritization in dual-task conditions. It remains difficult to establish a definitive causal relationship, and subsequent research should involve a randomized controlled trial to further clarify the effects of Tai Chi practice. Secondly, we observed that elderly Tai Chi practitioners exhibited a “posture first” phenomenon when responding to balance challenges, but this study lacked cognitive task performance. Additionally, while evidence suggests that elderly individuals using the “posture first strategy” in response to balance disturbances exhibit increased prefrontal cortical activation and arousal levels (Corp et al., 2018; Cleworth et al., 2019; Kan et al., 2025), this study also lacks the physiological indicators (e.g., heart rate variability, electrodermal activity, etc) to support such findings. Consequently, it remains unclear how these individuals prioritize tasks when addressing balance challenges involving cognitive load. Future research could use techniques such as near-infrared spectroscopy, electroencephalography and electrocardiography to explore the underlying mechanisms of Tai Chi on multisensory integration in dual-task conditions.

## 5 Conclusions

Our study revealed that elderly Tai Chi practitioners demonstrated enhanced postural stability in a dual-task paradigm using the modified Clinical Test of Sensory Interaction on Balance, which indicated superior multisensory integration with a concurrent cognitive task under sensory conflict. This offers new perspectives for expanding Tai Chi application in fall prevention interventions for the elderly.

## Data Availability

The raw data supporting the conclusions of this article will be made available by the authors, without undue reservation.
